# Structural Features and Defect Equilibrium in Cubic PrBa_1−*x*_Sr*_x_*Fe_2_O_6−*δ*_

**DOI:** 10.3390/ma15134390

**Published:** 2022-06-21

**Authors:** Ilia A. Leonidov, Alexey A. Markov, Mikhail A. Zavyalov, Oleg V. Merkulov, Elisaveta V. Shalaeva, Sergey S. Nikitin, Ekaterina V. Tsipis, Mikhail V. Patrakeev

**Affiliations:** 1 Institute of Solid State Chemistry, UB RAS, 620990 Ekaterinburg, Russia; leonidov@imp.uran.ru (I.A.L.); markov@ihim.uran.ru (A.A.M.); mazavyalov@gmail.com (M.A.Z.); shalaeva@ihim.uran.ru (E.V.S.); patrakeev@ihim.uran.ru (M.V.P.); 2 Osipyan Institute of Solid State Physics RAS, Moscow District, 142432 Chernogolovka, Russia; nikitins1997@gmail.com (S.S.N.); tsipis@issp.ac.ru (E.V.T.)

**Keywords:** perovskite, Sr-doped praseodymium-barium ferrite, incommensurate modulation, oxygen content, defect equilibrium, thermodynamics

## Abstract

The structure, oxygen non-stoichiometry, and defect equilibrium in perovskite-type PrBa_1−*x*_Sr*_x_*Fe_2_O_6−*δ*_ (*x* = 0, 0.25, 0.50) synthesized at 1350 °C were studied. For all compositions, X-ray diffraction testifies to the formation of a cubic structure (S.G. $Pm\bar{3}m$), but an electron diffraction study reveals additional diffuse satellites around each Bragg spot, indicating the primary incommensurate modulation with wave vectors about ±0.43$a^{*}$. The results were interpreted as a sign of the short-order in both A-cation and anion sublattices in the areas of a few nanometers in size, and of an intermediate state before the formation of an ordered superstructure. An increase in oxygen deficiency was found to promote the ordering, whereas partial substitution of barium by strontium caused the opposite effect. The oxygen content in oxides as a function of oxygen partial pressure and temperature was measured by coulometric titration, and the data were used for the modeling of defect equilibrium in oxides. The simulation results implied oxygen vacancy ordering in PrBa_1−*x*_Sr*_x_*Fe_2_O_6−*δ*_ that is in agreement with the electron diffraction study. Besides oxidation and charge disproportionation reactions, the reactions of oxygen vacancy distribution between non-equivalent anion positions, and their trapping in clusters with Pr^3+^ ions were taken into account by the model. It was demonstrated that an increase in the strontium content in Pr_0.5_Ba_0.5−*x*_Sr*_x_*FeO_3−*δ*_ suppressed ordering of oxygen vacancies, increased the binding energy of oxygen ions in the oxides, and resulted in an increase in the concentration of p-type carriers.

## 1. Introduction

The perovskite-type ferrites *R_x_A*_1−*x*_FeO_3−*δ*_, where *R* is a rare-earth element and *A* is an alkaline earth one, are known to possess mixed oxygen-ion and electron conductivity (MIEC), which makes them promising functional materials for electrochemical devices for energy conversion and storage, such as solid oxide fuel cells (SOFCs), membrane reactors for oxygen separation from air and synthesis gas production [1,2,3,4,5,6].

Ion and electron conductivity of p- and n-type are known to be strongly affected by the oxygen content in the oxide (3−*δ*), temperature, type and fraction of the *R* cation [7,8,9,10]. Although in the most MIEC ferrites *R_x_A*_1−*x*_FeO_3−*δ*_ lanthanum is used as *R*, materials based on perovskite-type ferrites with other *R* cations attracted much attention in recent years [6]. For example, the PrBaFe_2–*x*_Co*_x_*O_5+*δ*_ oxides were successfully tested as cathode materials for intermediate-temperature SOFCs [11]. *R*BaFe_2_O_5__+__δ_ ferrites were reported to exhibit high stability under reducing atmosphere at elevated temperatures [12]. PrBaFe_2_O_5+*δ*_ was shown to demonstrate good electro-catalytic activity and chemical stability in the H_2_ atmosphere [13]. This allows considering PrBaFe_2_O_5+*δ*_ based oxides as promising SOFCs anode materials [14,15,16].

A specific feature of Pr_0.5_Ba_0.5_FeO_3−*δ*_, in comparison with La_0.5_Ba_0.5_FeO_3−*δ*_, is a greater difference in the size of A-site residing cations, which should favor their ordering. Although a number of studies reported about Pr_0.5_Ba_0.5_FeO_3−*δ*_ (or PrBaFe_2_O_6−*δ*_) with a cubic perovskite structure [17,18], it was demonstrated that perfectly ordered double perovskite PrBaFe_2_O_5_ with an orthorhombic (SG *Pmmm*) structure could be synthesized via a special route under reducing atmosphere and appropriate temperature conditions [19]. Moreover, a literature analysis demonstrates that simple synthesis in air can also result in the formation of PrBaFe_2_O_6−*δ*_ with different lattice symmetry. Besides the cubic structure [15,17,20], a tetragonal one with a *P*4*/mmm* space group [14,21], and orthorhombic one with *Pmmm* [22,23] are also reported for this composition prepared in air. A comparison of the synthesis conditions used in the above mentioned studies demonstrates that the oxides with low symmetry were obtained at relatively low temperatures—950 or 1000 °C [14,21,22,23], whereas synthesis at 1330 or 1350 °C provides the formation of PrBaFe_2_O_6−*δ*_ with a cubic structure [15,17,20]. This feature shows that although Pr^3+^ and Ba^2+^ cations in ferrites tend to be ordered due to a considerable size difference, synthesis at high temperature ensures the formation of a substantially disordered state in the A sublattice. It is natural to expect that the degree of ordering can affect the characteristics of charge transport in PrBaFe_2_O_6−*δ*_. This should be taken into account in order to achieve reproducible operational properties of materials in electrochemical devices.

This work is aimed at studying the defect formation in PrBa_1−*x*_Sr*_x_*Fe_2_O_6−*δ*_. The interest in strontium substitution is attributed to its reported favorable impact on the electrical conductivity under both reducing and oxidizing conditions [15]. In order to minimize the uncertainties caused by possible structural ordering, the synthesis of oxides was performed at 1350 °C, which was demonstrated to provide formation of the cubic PrBaFe_2_O_6−*δ*_ structure. Since ordering on a scale below the XRD sensitivity limit but affecting the thermodynamic and transport properties of materials cannot be excluded, electron diffraction was used for the local structure examination. In the results of the study, it was found that the PrBaFe_2_O_6−*δ*_ oxide consists of the ordered domains with the size of a few nanometers. Due to a strong disposition to oxygen coordination less than 12-fold, Pr^3+^ ions in the ordered domains trap oxygen vacancies in steady clusters [Pr^3+^–V_O3_] that favors reduced oxygen content in oxide. The substitution of barium with strontium was found to promote the disordering of cation sublattice that in turn causes the decomposition of [Pr^3+^–V_O3_] clusters and release of oxygen vacancies, an increase in oxygen content and concomitant increase in p-type carrier concentration. All of these effects are expected to be favorable for the enhanced electrode performance.

## 2. Experimental Section

The PrBa_1−*x*_Sr*_x_*Fe_2_O_6−*δ*_ (*x* = 0, 0.25, 0.50) ferrites were prepared by the glycine-nitrate combustion synthesis. Metal iron, praseodymium oxide, high purity barium and strontium carbonates taken in the necessary proportion were dissolved in nitric acid. Glycine was added to the obtained solution in a 50% excess to nitrates, after which the mixture was stirred and heated until self-ignition occurred. The combustion product was annealed at 900 °C for 10 h in order to remove organic and carbon residues. Then, the product was milled, pressed into discs 20 mm in diameter and about 2 mm thick, and sintered at 1350 °C for 10 h. The obtained samples were milled into powder to be used in the further study. In order to evaluate material stability under reducing conditions, the samples were held in a gas mixture of 89%Ar-1%CO-10%CO_2_ at 950 °C ($p_{O_{2}}$~10^−15^ atm) for 5 h and then cooled down at a rate of 5°/min for structural analysis.

The phase purity of the materials and their structural characteristics were examined by X-ray diffraction (XRD) on a Shimadzu XRD–7000 diffractometer with CuK_α_ radiation. The analysis of XRD data was performed by the Rietveld refinement method using the GSAS-II software [24].

The local structure of PrBa_1−*x*_Sr*_x_*Fe_2_O_6−*δ*_ was studied by electron diffraction with a JEM-200 CX microscope. Powder specimens of oxides were treated in an isobutyl alcohol bath with ultrasonic stirring, and then used to form sediments upon a carbon film. The deposited film was supported with a copper grid.

The oxygen content in oxides was measured in relation to temperature by a Setaram TG-92 thermal analyzer in air. The measurements were performed in a cooling mode at a rate of 1 °/min after preliminary sample exposure at 950 °C for 5 h. The oxygen content in PrBa_1−*x*_Sr*_x_*Fe_2_O_6−*δ*_ as a function of oxygen partial pressure ($p_{O_{2}}$) at different temperatures was measured by coulometric titration. Measurements at oxygen partial pressure above 10^−3^ atm were carried out in the isothermal mode by a stepwise decrease in $p_{O_{2}}$ and subsequent holding for sample equilibration with the gas phase. Below 10^−3^ atm, a step temperature mode was used for measurements because it provides a faster equilibrium state achievement [25]. The experimental data were recorded only when a variation in the logarithm of the oxygen partial pressure over the sample became less than 0.01 per hour. This equilibrium criterion provided good reversibility of measurement results. More details related to the experimental setup and measurement conditions can be found elsewhere [26,27].

## 3. Data Analysis Methods

### 3.1. Model of a Point Defect Equilibrium

In the studied ranges of temperature and partial pressure of oxygen, the iron in PrBa_1−*x*_Sr*_x_*Fe_2_O_6−*δ*_ can be in ^2+^, ^3+^ and ^4+^ oxidation states. Therefore, the composition of a solid solution can be presented as ${\mathrm{Pr}}^{3+}{\mathrm{Ba}}_{1-x}^{2+}{\mathrm{Sr}}_{x}^{2+}{\mathrm{Fe}}_{n}^{2+}{\mathrm{Fe}}_{a}^{3+}{\mathrm{Fe}}_{p}^{4+}O_{6-\delta }^{2-}$.

**Figure 1:**
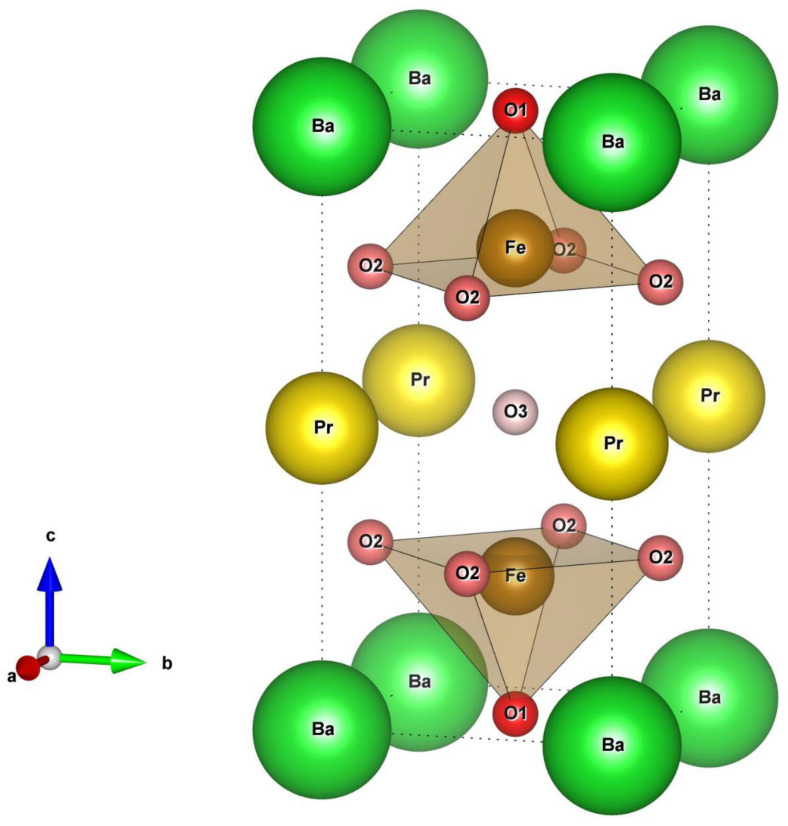
Unit cell of double perovskite PrBaFe_2_O_6−*δ*_. O3 position has the lowest occupancy.

Taking into account the disposition of barium and praseodymium to ordering, it is assumed that irrespective of the results of the XRD analysis, the oxides can have an ordered double perovskite structure at a low scale, as shown in Figure 1. There are three oxygen positions in double perovskite: O1, O2 and O3 with different bond energy. As in the case of PrBaCo_2_O_6−*δ*_, the O1 position is considered to be completely occupied with oxygen ions, unlike O3, where the probability of vacancy appearance is the highest. Besides, the formation of oxygen vacancies in the O2 position is also possible [28].

The oxygen partial pressure in the gas phase affects the oxygen content in the oxides via the oxidation reaction:

(1) $$\frac{1}{2}O_{2}+V_{O3}+2{\mathrm{Fe}}^{3+}⇄O_{O3}^{2-}+2{\mathrm{Fe}}^{4+}\;K_{\mathrm{ox}}=\frac{\left[O_{O3}^{2-}\right]{\left[{\mathrm{Fe}}^{4+}\right]}^{2}}{p_{O_{2}}^{\frac{1}{2}}\left[V_{O3}\right]{\left[{\mathrm{Fe}}^{3+}\right]}^{2}}$$

where $V_{O3}$ designates oxygen vacancy at the O3 site. The charge disproportionation reaction on iron ions can also contribute to defect formation:

(2) $${2\mathrm{Fe}}^{3+}⇄{\mathrm{Fe}}^{4+}+{\mathrm{Fe}}^{2+}\;K_{d}=\frac{\left[{\mathrm{Fe}}^{2+}\right]\left[{\mathrm{Fe}}^{4+}\right]}{{\left[{\mathrm{Fe}}^{3+}\right]}^{2}}$$

The redistribution of oxygen ions between the O2 and O3 sites is assumed to be possible:

(3) $$O_{O2}^{2-}+V_{O3}^{}⇄O_{O3}^{2-}+V_{O2}^{}\;K_{\mathrm{od}}=\frac{\left[O_{O3}^{2-}\right]\left[V_{O2}^{}\right]}{\left[O_{O2}^{2-}\right]\left[V_{O3}^{}\right]}$$

It should be noted that in contrast to La^3+^, Ba^2+^ or Sr^2+^, which can have a 12-fold oxygen coordination necessary for the cubic perovskite structure, for Pr^3+^ ion, the maximum coordination number is known to be 9 [29]. This should restrict the number of oxygen ions around praseodymium and cause the trapping of oxygen vacancies in clusters:

(4) $${\mathrm{Pr}}^{3+}+V_{O3}⇄[{\mathrm{Pr}}^{3+}-V_{O3}]\;K_{c}=\frac{\left[{\mathrm{Pr}}^{3+}-V_{O3}^{}\right]}{\left[{\mathrm{Pr}}^{3+}\right]\left[V_{O3}^{}\right]}$$

The temperature dependences of the equilibrium constants of these reactions are governed by the well-known thermodynamic expression:

(5) $$K_{i}=\exp\left(-\frac{\Delta G_{i}^{0}}{RT}\right)=\exp\left(-\frac{\Delta H_{i}^{0}}{RT}+\frac{\Delta S_{i}^{0}}{R}\right)$$

where R is the gas constant, and $\Delta G_{i}^{0}$, $\Delta H_{i}^{0}$ and $\Delta S_{i}^{0}$ are the standard Gibbs free energy, enthalpy and entropy changes for the defect formation reactions, respectively. Additionally, the requirement of site conservation in the iron sublattice and the electroneutrality condition should be taken into account:

(6) $$\left[{\mathrm{Fe}}^{2+}\right]+\left[{\mathrm{Fe}}^{3+}\right]+\left[{\mathrm{Fe}}^{4+}\right]=2$$

(7) $$\left[{\mathrm{Pr}}^{3+}\right]+\left[{\mathrm{Pr}}^{3+}-V_{O3}^{}\right]=1$$

(8) $$\left[V_{O2}^{}\right]+\left[O_{O2}^{2-}\right]=4$$

(9) $$\left[V_{O3}^{}\right]+\left[O_{O3}^{2-}\right]+\left[{\mathrm{Pr}}^{3+}-V_{O3}^{}\right]=1$$

(10) $$\left[V_{O2}^{}\right]+\left[V_{O3}^{}\right]+\left[{\mathrm{Pr}}^{3+}-V_{O3}^{}\right]=\delta$$

(11) $$5+2\left[{\mathrm{Fe}}^{2+}\right]+3\left[{\mathrm{Fe}}^{3+}\right]+4\left[{\mathrm{Fe}}^{4+}\right]=2\left(6-\delta \right)$$

Preliminary calculations for the PrBaFe_2_O_6−*δ*_ demonstrated that the vacancy concentration in the O2 position does not exceed 10^−9^ over the studied ranges of temperature and partial pressure of oxygen. Therefore, Equations (3) and (8) were excluded from further consideration for this composition.

The joint solution of Equations (1)–(11) allows one to combine the partial pressure of oxygen, the oxygen content in oxides, and the thermodynamic parameters of reactions (1)–(4) in the single equation omitted here due to complexity and space limitation.

### 3.2. Partial Molar Enthalpy and Entropy of Oxygen in Pr_0.5_Ba_0.5−__x_Sr_x_FeO_3−__δ_

The thermodynamic equilibrium between the oxide and the surrounding gas phase implies that the respective chemical potentials $\mu_{O}$ and $\mu_{O_{2}}$ are equal:

(12) $$\frac{1}{2}\mu_{O_{2}}=\mu_{O}$$

The chemical potential of oxygen in the gas phase is determined as follows:

(13) $$\mu_{O_{2}}=\mu_{O_{2}}^{ο}+RT\ln\left(p_{O_{2}}\right)$$

where $\mu_{O_{2}}^{ο}$ is the chemical potential of oxygen in the gas phase under standard conditions. Thus, the chemical potential of oxygen in oxides relative to the standard state in the gas phase $\Delta \mu_{O}$ can be expressed as:

(14) $$\Delta \mu_{O}=\mu_{O}-\frac{1}{2}\mu_{O_{2}}^{ο}=\frac{1}{2}RT\ln p_{O_{2}}$$

In turn, this chemical potential of oxygen in oxides is related to the respective partial molar enthalpy Δ*h*_O_ and entropy Δ*s*_O_:

(15) $$\Delta \mu_{O}=\Delta h_{O}-T\Delta s_{O}$$

The Gibbs–Helmholtz relations can be obtained from Equations (9) and (10):

(16) $$\Delta h_{O}=\frac{R}{2}{\left[\partial \left(\ln\left(p_{O_{2}}\right)\right)/\partial \left(1/T\right)\right]}_{\delta }$$

(17) $$\Delta s_{O}=-\frac{R}{2}{\left[\partial \left(T\cdot \ln\left(p_{O_{2}}\right)\right)/\partial \left(T\right)\right]}_{\delta }$$

### 3.3. Statistical Thermodynamic Modeling

The total Gibbs energy for the PrBa_1−*x*_Sr*_x_*Fe_2_O_6−*δ*_ solid solution is determined, as follows:

(18) $$G=G^{0}+\left[{\mathrm{Pr}}^{3+}\right]\mu_{{\mathrm{Pr}}^{2+}}^{0}+\left[{\mathrm{Pr}}^{3+}-V_{O3}\right]\mu_{{\mathrm{Pr}}^{3+}-V_{O3}}^{0}+\left(1-x\right)\cdot \mu_{{\mathrm{Ba}}^{2+}}^{0}+x\cdot \mu_{{\mathrm{Sr}}^{2+}}^{0}+\left[{\mathrm{Fe}}^{2+}\right]\mu_{{\mathrm{Fe}}^{2+}}^{0}\;+\left[{\mathrm{Fe}}^{3+}\right]\mu_{{\mathrm{Fe}}^{3+}}^{0}+\left[{\mathrm{Fe}}^{4+}\right]\mu_{{\mathrm{Fe}}^{4+}}^{0}+\left[O_{O2}^{2-}\right]\mu_{O_{O2}^{2-}}^{0}+\left[O_{O3}^{2-}\right]\mu_{O_{O3}^{2-}}^{0}+\left[V_{O2}^{}\right]\mu_{V_{O2}^{}}^{0}+\left[V_{O3}^{}\right]\mu_{V_{O3}^{}}^{0}-T\cdot S_{\mathrm{conf}}$$

where $G^{0}$ is the standard Gibbs energy, $\mu^{0}$ is the chemical potential of the respective component and $S_{\mathrm{conf}}$ is the total configurational entropy of the solid solution.

The configurational entropy in the Stirling approximation is defined by the formula [30]:

(19) $$S_{\mathrm{conf}}=-R\cdot \sum_{i}x_{i}\ln\left(x_{i}\right)$$

where $x_{i}$ is the content of i-th component (Pr^3+^, [${\mathrm{Pr}}^{3+}-V_{O3}^{}$], Ba^2+^, Sr^2+^, Fe^2+^, Fe^3+^, Fe^4+^, $V_{O2}^{}$, $O_{O2}^{2-}$, $V_{O3}^{}$ and $O_{O3}^{2-}$).

The chemical potential of oxygen can be determined as:

(20) $$\mu_{O}=\frac{\partial G}{\partial \left[O^{2-}\right]}=\frac{\partial G}{\partial \left(6-\delta \right)}=-\frac{\partial G}{\partial \delta }=-\frac{\partial \left[{\mathrm{Pr}}^{3+}\right]}{\partial \delta }\cdot \mu_{{\mathrm{Pr}}^{3+}}^{0}-\frac{\partial \left[{\mathrm{Pr}}^{3+}-V_{O3}\right]}{\partial \delta }\cdot \mu_{{\mathrm{Pr}}^{3+}-V_{O3}}^{0}-\;-\frac{\partial \left[{\mathrm{Fe}}^{2+}\right]}{\partial \delta }\cdot \mu_{{\mathrm{Fe}}^{2+}}^{0}-\frac{\partial \left[{\mathrm{Fe}}^{3+}\right]}{\partial \delta }\cdot \mu_{{\mathrm{Fe}}^{3+}}^{0}-\frac{\partial \left[{\mathrm{Fe}}^{4+}\right]}{\partial \delta }\cdot \mu_{{\mathrm{Fe}}^{4+}}^{0}-\frac{\partial \left[V_{O2}^{}\right]}{\partial \delta }\cdot \mu_{V_{O2}^{}}^{0}-\;-\frac{\partial \left[O_{O2}^{2-}\right]}{\partial \delta }\cdot \mu_{O_{O2}^{2-}}^{0}-\frac{\partial \left[V_{O3}^{}\right]}{\partial \delta }\cdot \mu_{V_{O3}^{}}^{0}-\frac{\partial \left[O_{O3}^{2-}\right]}{\partial \delta }\cdot \mu_{O_{O3}^{2-}}^{0}-T\cdot s_{O\;\mathrm{conf}}$$

(21) $$s_{O\;\mathrm{conf}}=-\frac{\partial S_{\mathrm{conf}}}{\partial \delta }$$

Using Equations (1)–(11), (20) and (21), one can obtain:

(22) $$\Delta \mu_{O}=\left(\Delta G_{\mathrm{ox}}-\Delta G_{c}\right)-\frac{\partial \left[V_{O2}\right]}{\partial \delta }\cdot \left(\Delta G_{c}-\Delta G_{\mathrm{od}}\right)+\frac{\partial \left[V_{O3}\right]}{\partial \delta }\cdot \Delta G_{c}-\;-\frac{\partial \left[{\mathrm{Fe}}^{2+}\right]}{\partial \delta }\cdot \Delta G_{d}-T\cdot s_{O\;\mathrm{conf}}$$

(23) $$\Delta h_{O}=\left(\Delta H_{\mathrm{ox}}-\Delta H_{c}\right)-\frac{\partial \left[V_{O2}\right]}{\partial \delta }\cdot \left(\Delta H_{c}-\Delta H_{\mathrm{od}}\right)+\frac{\partial \left[V_{O3}\right]}{\partial \delta }\cdot \Delta H_{c}-\frac{\partial \left[{\mathrm{Fe}}^{2+}\right]}{\partial \delta }\cdot \Delta H_{d}$$

(24) $$\Delta s_{O}=\left(\Delta S_{\mathrm{ox}}-\Delta S_{c}\right)-\frac{\partial \left[V_{O2}\right]}{\partial \delta }\cdot \left(\Delta S_{c}-\Delta S_{\mathrm{od}}\right)+\frac{\partial \left[V_{O3}\right]}{\partial \delta }\cdot \Delta S_{c}-\;-\frac{\partial \left[{\mathrm{Fe}}^{2+}\right]}{\partial \delta }\cdot \Delta S_{d}-T\cdot s_{O\;\mathrm{conf}}$$

(25) $$s_{O\;\mathrm{conf}}=R\cdot \left(\begin{array}{l} \frac{\partial \left[V_{O2}\right]}{\partial \delta }\cdot \left(\ln\left(\frac{K_{\mathrm{od}}}{K_{c}}\right)+1\right)+\frac{\partial \left[V_{O3}\right]}{\partial \delta }\cdot \left(\ln\left(\frac{1}{K_{c}}\right)+1\right)\\ +\frac{\partial \left[{\mathrm{Fe}}^{2+}\right]}{\partial \delta }\cdot \ln\left(K_{d}\right)+\ln\left(\frac{\left[{\mathrm{Pr}}^{3+}-V_{O3}^{}\right]\cdot {\left[{\mathrm{Fe}}_{}^{3+}\right]}^{2}}{{\left[{\mathrm{Fe}}^{4+}\right]}^{2}\cdot \left[O_{O3}^{}\right]\cdot \left[{\mathrm{Pr}}^{3+}\right]}\right)-1 \end{array}\right)$$

The detailed derivation of Equations (22)–(25) and expressions for $\frac{\partial \left[V_{O2}\right]}{\partial \delta }$, $\frac{\partial \left[V_{O3}\right]}{\partial \delta }$, and $\frac{\partial \left[{\mathrm{Fe}}^{2+}\right]}{\partial \delta }$ are omitted because of their complexity and space limitation.

## 4. Results and Discussion

### 4.1. Structure and Oxygen Content

**Figure 2:**
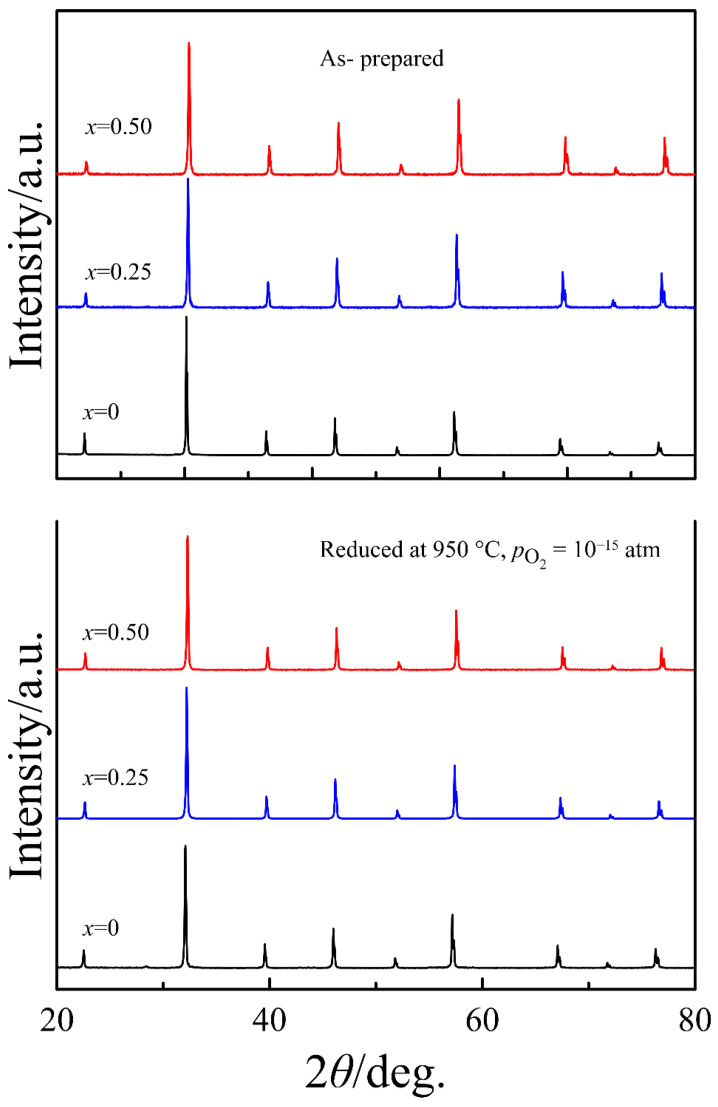
Room-temperature X-ray powder diffraction patterns of PrBa_1−*x*_Sr*_x_*Fe_2_O_6−*δ*_ for as-prepared samples and samples reduced at $p_{O_{2}}$ = 10^−15^ atm.

The XRD patterns of the PrBa_1−*x*_Sr*_x_*Fe_2_O_6−*δ*_ samples, as-prepared in air and subjected to reductive treatment at 950 °C and presented in Figure 2, indicate that all oxides are single-phase with a cubic perovskite-type structure (S.G. $Pm\bar{3}m$). This demonstrates that the disordered state of the A sublattice provided by synthesis at high temperature is steady and cannot be notably modified by reductive treatment at 950 °C. The unit cell parameter of 3.9387(1) Å for the as-prepared Pr_0.5_Ba_0.5_FeO_3−*δ*_ is in reasonable agreement with 3.930 and 3.934 Å, which were reported earlier [17,20].

**Figure 3:**
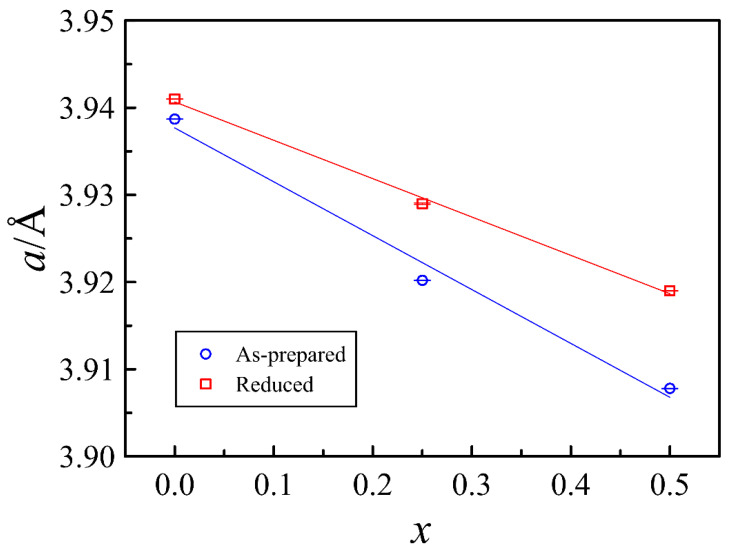
Unit cell parameter of PrBa_1−*x*_Sr*_x_*Fe_2_O_6−*δ*_ after different thermal treatments as a function of strontium content.

A decrease in the lattice parameter with increasing strontium content observed in Figure 3 for both as-prepared and reduced series of oxides is naturally attributed to the smaller size of Sr^2+^ ions than that of Ba^2+^. The larger lattice parameter of the reduced oxides is explained by higher fractions of Fe^3+^ (R_CN6_ = 0.645 Å) and Fe^2+^ (R_CN6_ = 0.75 Å) ions with a larger radius than of Fe^4+^ (R_CN6_ = 0.585 Å) [29]. Some deviation of the lattice parameter dependence on the strontium content from linear behavior should be attributed to the non-trivial relation of 3−*δ* vs. *x*.

**Figure 4:**
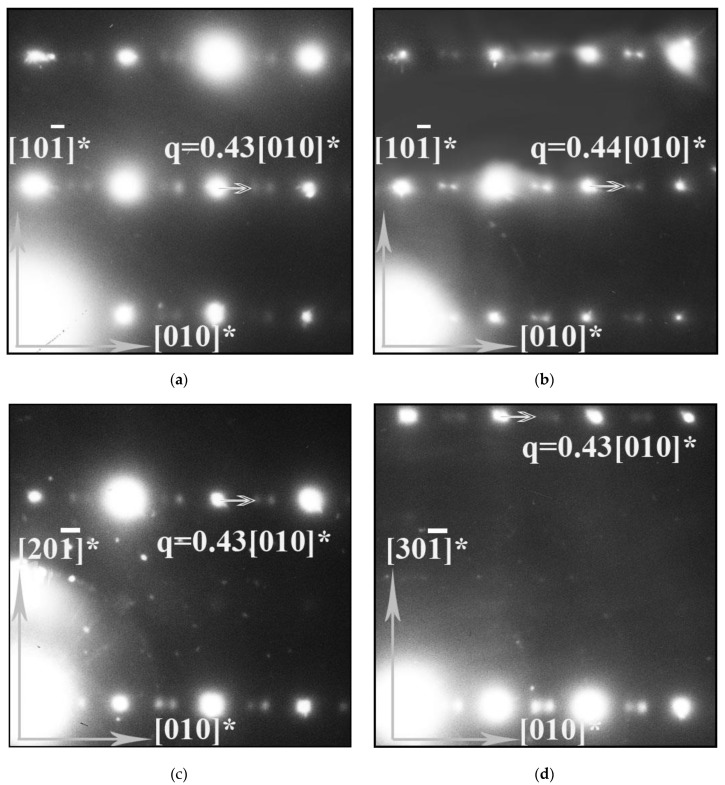
SAED patterns for the reduced PrBaFe_2_O_6−*δ*_, zone axis: (**a**,**b**)—[101], (**c**)—[102], (**d**)—[103]. Positions of the diffuse reflections are shown by the incommensurate ***q*** vectors.

**Figure 5:**
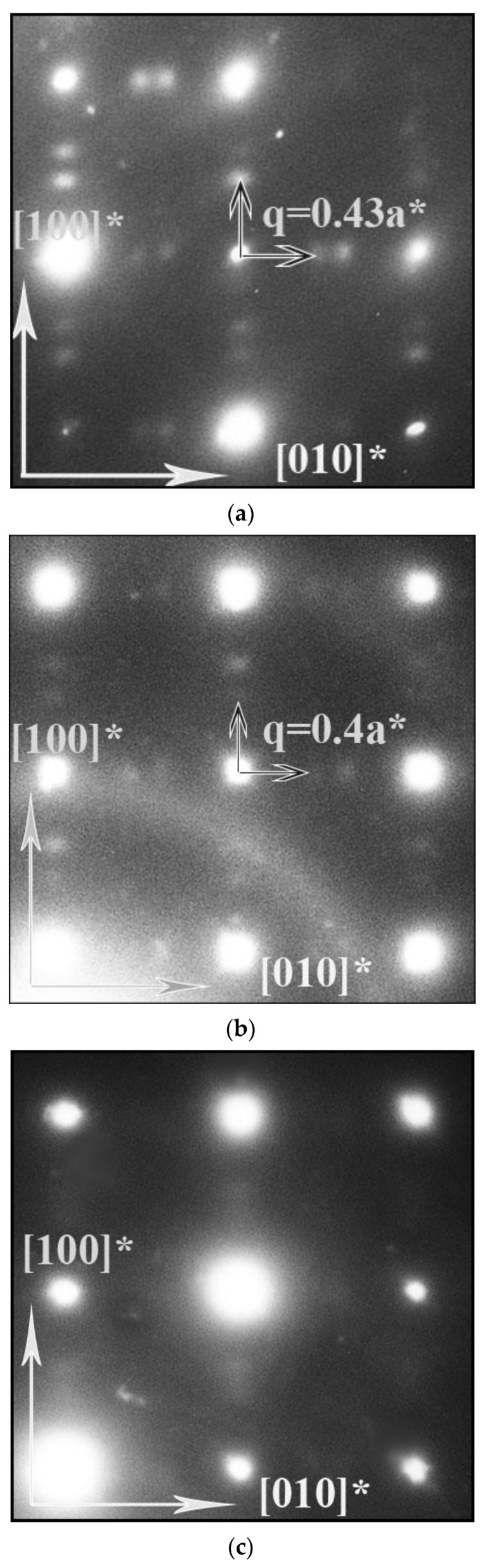
[001] SAED patterns of PrBaFe_2_O_6−*δ*_ in (**a**)—reduced, (**b**)—as-prepared state. (**c**)—[001] SAED pattern for as-prepared PrBa_0.5_Sr_0.5_Fe_2_O_6−*δ*_. Positions of the diffuse reflections are shown by the incommensurate ***q*** vectors.

An electron-diffraction (ED) study was carried out to investigate the local structure features of PrBaFe_2_O_6−*δ*_ and the strontium-substituted derivative. For all SAED (selected area electron diffraction) patterns, the observed Bragg reflections correspond to a primitive cubic structure ($Pm\bar{3}m$). For the reduced samples, in all SAED patterns with zone axes [001], [101], [102] and [103] et al., which contain reciprocal lattice directions of the [001]* type, additional diffuse satellites are observed around each Bragg spot at the positions given by wave vectors ***q*** (Figure 4 and Figure 5a). The average wave vectors are equal to ±0.43$a^{*}$ ($a^{*}$ is the unit cell vector of reciprocal cubic lattice) and hence, are incommensurate with the unit cell vectors of perovskite cubic structure. Some electron-diffraction patterns of individual crystals reveal sharper incommensurate satellites, with their wave vectors being slightly larger and close to 0.44$a^{*}$. For the as-prepared PrBaFe_2_O_6−*δ*_ samples, in the SAED patterns, the incommensurate satellites are weak and more blurred (Figure 5b). In addition, for the [001] SAED pattern, the positions of these satellites are given by the wave vectors ***q*** ≈ ±0.4$a^{*}.$ For as-prepared PrBa_1−*x*_Sr*_x_*Fe_2_O_6−*δ*_, the incommensurate satellites are barely distinguishable (Figure 5c).

Such incommensurate modulations are believed to be created by atomic displacement and/or an occupancy (composition) probability wave with wave vector ***q*** for some atomic sites in the lattice, and the modulated state can be intermediate before the formation of ordered superstructures with changes in the composition or temperature [31]. For a range of the A-sublattice-doped ferrites *R_x_*Sr_1−*x*_FeO_3−*δ*_ (*R* is Y, Ce), which are known to be prone to the formation of ordered superstructures, similar incommensurate modulations have been observed in previous TEM studies [32,33,34,35]. The modulated states and ordered phases were found mainly in the reduced samples. In the *R*BaFe_2_O_5+*w*_ ferrites, reduced at high temperatures, a few ordered phases with varying oxygen nonstoichiometry (*w*) were also recently obtained [12,36]. The oxygen-poor BaPrFe_2_O_5.01_ was reported to have an orthorhombic double-perovskite structure (S.G. *Pmmm*) at room temperature. For compositions with 0.1 < *w* < 0.48, the orthorhombic distortion is unstable, and a tetragonal superstructure *P*4/*mmm* is formed at temperatures above 1000 °C. At temperatures between 600 and 1000 °C and *w* ≥ 0.5 the presence of the Pr_2_Ba_2_Fe_4_O_11_ orthorhombic structure, *Cmmm* was confirmed [12]. Therefore, the observed incommensurate modulations in the reduced PrBaFe_2_O_6−*δ*_ can be considered as a sign of an intermediate state related to the order–disorder transformations in ferrite. A number of our experimental findings support the above assumption. The intensity of incommensurate satellites substantially decreases in the as-prepared PrBaFe_2_O_6−*δ*_ sample. In addition, Sr-doping of the as-prepared PrBaFe_2_O_6−*δ*_ sample leads to the almost complete disappearance of these satellites.

Similar to *R_x_*Sr_1−*x*_FeO_3−*δ*_ (*R* is Y, Ce), all PrBaFe_2_O_6−*δ*_ superstructures are characterized by simultaneous ordering in the oxygen-vacancy and A-metal sublattices. In the ordered Pr_2_Ba_2_Fe_4_O_11_ phase, the ordering of both sublattices (Ba/Pr and oxygen/vacancy) is accompanied by atom displacements from the ideal perovskite-like positions in the <100> directions of the basic cubic lattice [12]. According to the “modulation wave approach” [37], composition and displacive modulation waves with wave vectors ***q*** can be suggested in both sublattices of the reduced PrBaFe_2_O_6−*δ*_, with modulation waves being poorly correlated because only the first order (primary) but not the higher order (±n***q***) satellites are observed. Such a state with only primary incommensurate diffuse satellites was proposed to be called a disordered modulated structure, which does not exhibit a long-range order, only a short-range (local) order [38]. The correlation length, which is inversely proportional to the finite width (or smearing) of diffuse incommensurate peaks, can be estimated at about a few nanometers. Hence, the size of the region with the modulation wave is close to this value. This estimation is generally consistent with HRTEM data on the nonuniform nanosized structure of Y_0.25_Sr_0.75_FeO_3−*δ*_ powder demonstrating similar primary incommensurate diffuse peaks in the SAED pattern [32]. It can be observed in Figure 4 that the correlation length and ordering tendency decreases in the series of samples: reduced PrBaFe_2_O_6−*δ*_, as-prepared PrBaFe_2_O_6−*δ*_, and as-prepared PrBa_0.5_Sr_0.5_Fe_2_O_6−*δ*_. A decrease in value of the wave vector ***q*** is also observed with a decrease in the correlation length.

Thus, the electron diffraction study has found that in the reduced PrBaFe_2_O_6−*δ*_, a disordered modulated (or short-range) structure but not an ordered superstructure is formed. The short-range is characterized by primary incommensurate modulations with wave vectors ***q*** = ±0.43$a^{*}$ (***a*** is perovskite-lattice vector), which are considered to be related to displacement and composition waves in both Ba/Pr and oxygen/vacancy sublattices of perovskite structure. Oxygen nonstoichiometry, as well as Sr-doping, are crucial parameters of incommensurate modulations, correlation length and ordering tendency in Pr_0.5_Ba_0.5−*x*_Sr*_x_*FeO_3−*δ*_.

**Figure 6:**
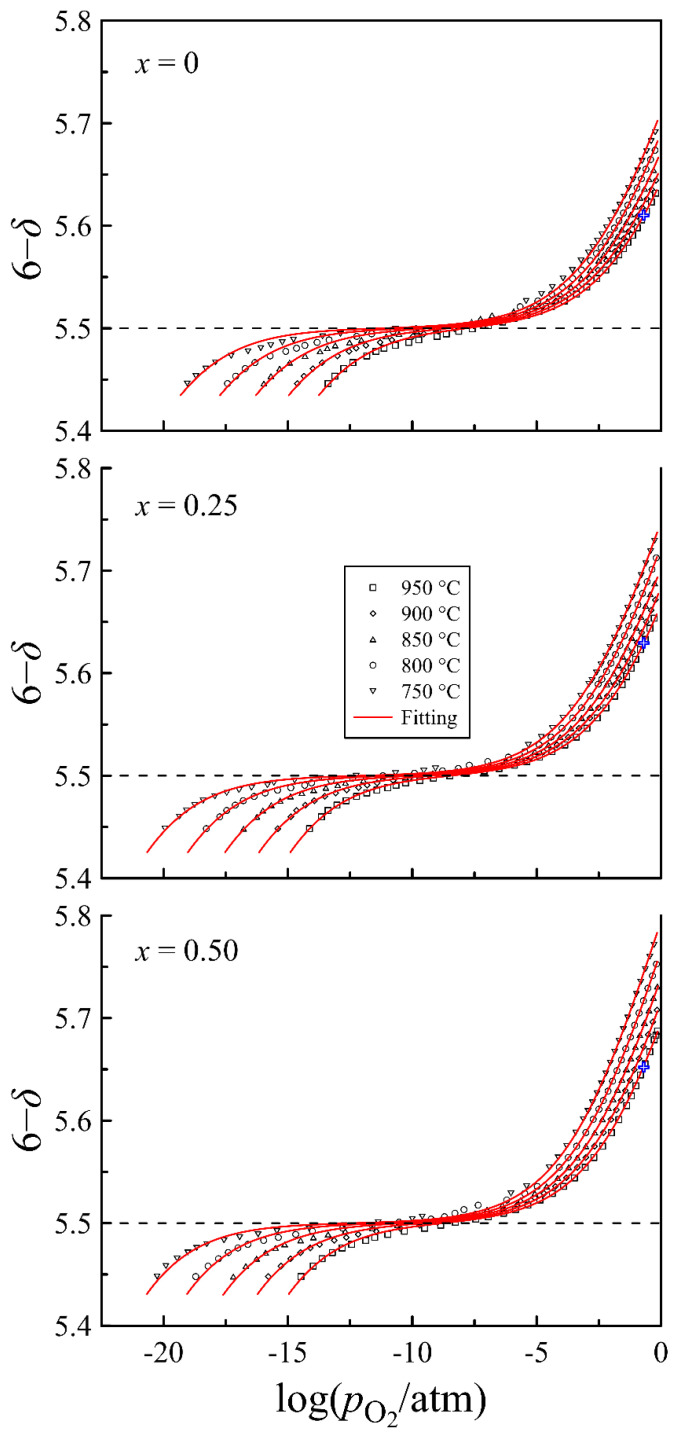
Oxygen content in PrBa_0.5_Sr_0.5_Fe_2_O_6−*δ*_ as a function of oxygen partial pressure at different temperatures. Dashed lines indicate the oxygen content corresponding to the nominal iron oxidation state in 3+ oxides. Solid lines represent the results of the model calculation. The cross-like symbols designate oxygen content at $p_{O_{2}}$ = 0.21 atm and 950 °C.

**Figure 7:**
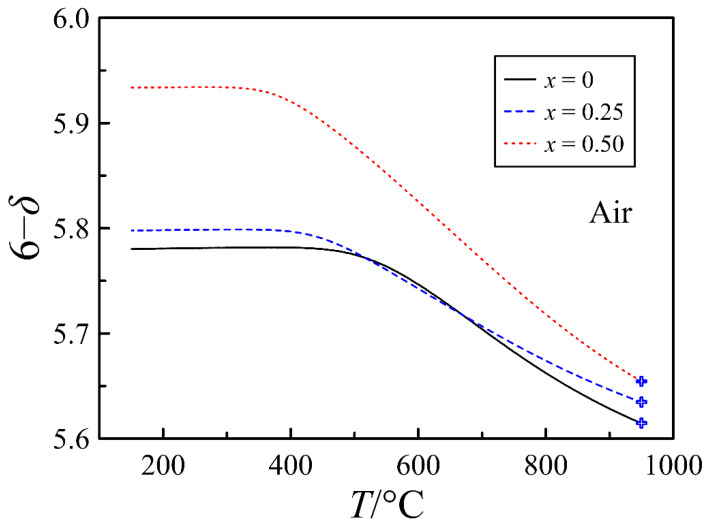
Oxygen content in PrBa_1−*x*_Sr*_x_*Fe_2_O_6−*δ*_ versus temperature, calculated from thermogravimetric results in air. The cross-like symbols indicate reference values of oxygen content taken from the data in Figure 6.

Figure 6 presents isothermal plots of the oxygen content in PrBa_1−*x*_Sr*_x_*Fe_2_O_6−*δ*_ versus the oxygen partial pressure at different temperatures. All isotherms contain a wide plateau with an inflection point, which corresponds to the nominal oxidation state 3+ of iron according to the formula ${\mathrm{Pr}}_{}^{3+}Ba_{1-x}^{2+}Sr_{x}^{2+}Fe_{2}^{3+}O_{5.5}^{2-}$. These features were used to adjust the experimental arrays $p_{O_{2}}$–*T*–(6−*δ*) to the reference value of oxygen content (6−*δ*) = 5.5. The cross-like symbols on the 950 °C isotherms in Figure 6 designate the oxygen content in the oxides in air. These values indicated by the same symbols in Figure 7 were used as references to calculate oxygen content data from thermogravimetric measurement results.

As can be observed in Figure 7, all compositions at room temperature are oxygen deficient. This deficiency increases with increasing barium content and is presumably attributed to the ordering of oxygen vacancies accompanied by the formation of stable $\left[{\mathrm{Pr}}^{3+}-V_{O3}^{}\right]$ clusters. The maximum oxygen content, which PrBaFe_2_O_6−*δ*_ attains at slow cooling in air, is 5.78, which is in good agreement with the doubled value of ~2.885 determined for Pr_0.5_Ba_0.5_FeO_3−*δ*_ in [14]. Another effect of strontium substitution, which Figure 7 reveals, is a decrease in the onset of the oxygen release temperature. This effect most probably reflects the improvement of oxygen exchange kinetics.

### 4.2. Defect Equilibrium Parameters and Thermodynamic Quantities

The experimental data on the oxygen content in PrBa_1−*x*_Sr*_x_*Fe_2_O_6−*δ*_, versus the oxygen partial pressure at different temperatures presented in Figure 6 by dots, were employed for the modeling of defect equilibrium in the oxides. The results of calculations presented in Figure 6 by solid lines correspond reasonably well to the experimental data. The obtained thermodynamic parameters of the defect formation reactions are summarized in Table 1.

The enthalpy of the charge disproportionation reaction (2) is similar for all compositions of PrBa_1−*x*_Sr*_x_*Fe_2_O_6−*δ*_. In contrast, the enthalpy of the oxidation reaction (1) decreases with increasing strontium content in PrBa_1−*x*_Sr*_x_*Fe_2_O_6−*δ*_. The same trend in Δ*H*_ox_ variation was observed for La_0.49_Ba_0.5−*x*_Sr*_x_*FeO_3−*δ*_ [39]. A decrease in Δ*H*_ox_ upon an increase in strontium content indicates an increase in the binding energy of oxygen in the oxide, which to some degree can result from lattice shrinkage and the respective contraction of the Fe-O bond lengths. At the same time, a big difference in the values of Δ*H*_ox_ for La_0.49_Ba_0.5_FeO_3−*δ*_ (−95 kJmol^−1^) and PrBaFe_2_O_6−*δ*_ (−80 kJmol^−1^) ferrites clearly indicates the presence of other factors affecting the enthalpy of the oxidation reaction. The origin of this difference can be related to lower available oxygen coordination for Pr^3+^ ions in comparison to La^3+^. It is easy to estimate that the average 9-fold oxygen coordination of Pr^3+^ ions in PrBaFe_2_O_6−*δ*_ at the full 12-fold coordination of Ba^2+^ ions corresponds to the oxygen content of 5.25, which is less than the experimental results in Figure 6. One can assume that excessive oxygen around the Pr^3+^ ion is less favorable for this oxide and its incorporation results in a decrease in the binding energy of oxygen ions reflecting a higher $\Delta H_{ox}^{ο}$ value, in comparison to La_0.49_Ba_0.5_FeO_3−*δ*_ with 12-fold oxygen coordination for La^3+^ ions.

Another interesting trend that the data in Table 1 demonstrate is an increase in the Δ*H*_c_ with an increase in strontium content in PrBa_1−*x*_Sr*_x_*Fe_2_O_6−*δ*_ indicating that strontium substitution favors the release of V_O3_ oxygen vacancies from $\left[{\mathrm{Pr}}^{3+}-V_{O3}^{}\right]$ clusters.

**Figure 8:**
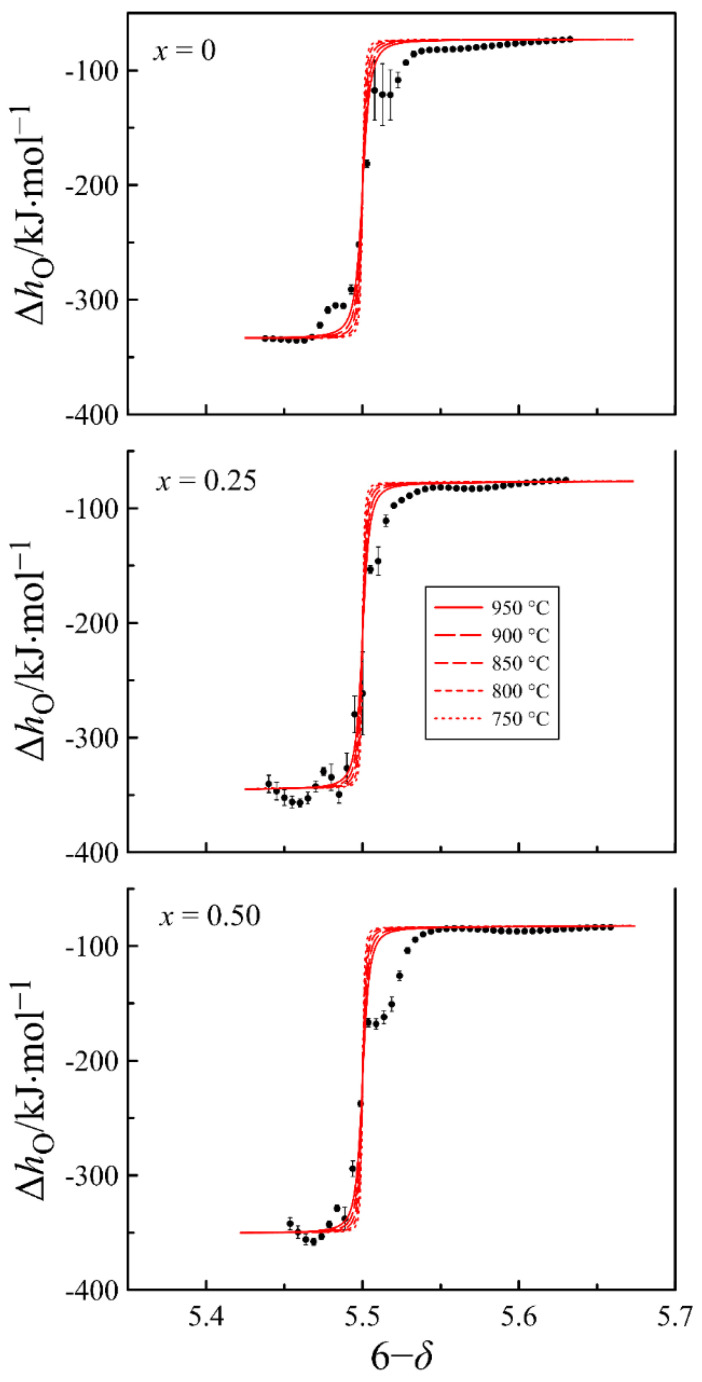
Partial molar enthalpy of oxygen in PrBa_1−*x*_Sr*_x_*Fe_2_O_6−*δ*_ as a function of oxygen content in the oxides. Dots show the results obtained with the Gibbs–Helmholtz relation (16). Lines represent the results of statistical thermodynamic calculations with Equation (23).

**Figure 9:**
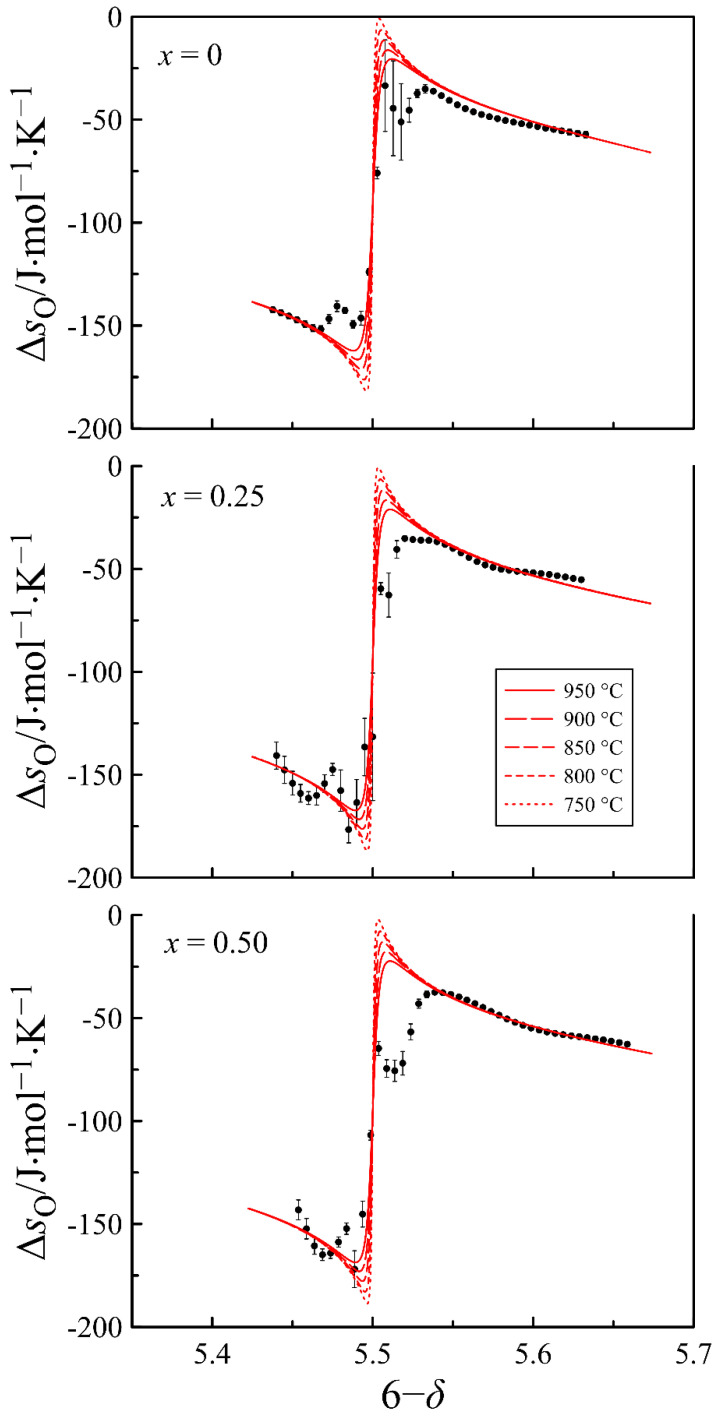
Partial molar enthalpy entropy of oxygen in PrBa_1−*x*_Sr*_x_*Fe_2_O_6−*δ*_ as a function of oxygen content in the oxide. Dots show the results obtained using the Gibbs–Helmholtz relation (17). Lines represent the results of statistical thermodynamic calculations with Equation (24).

In order to verify the adequacy of the used point defect model and the obtained thermodynamic parameters, the partial molar enthalpy and entropy of oxygen versus the oxygen content in the oxides are calculated and shown in Figure 8 and Figure 9, respectively. The dots in the figures present the results obtained directly from the experimental data via Gibbs–Helmholtz relations (16) and (17), whereas the lines show the results of statistical thermodynamic calculations using Equations (23) and (24).

According to Equation (23), partial molar enthalpy of oxygen at high oxygen content should approach the $\Delta H_{ox}^{ο}-\Delta H_{c}^{ο}$ value, corresponding to the enthalpy of the $\frac{1}{2}O_{2}+2{\mathrm{Fe}}^{3+}+\left[{\mathrm{Pr}}^{3+}-V_{O3}^{}\right]⇄O_{O3}^{2-}+2{\mathrm{Fe}}^{4+}+{\mathrm{Pr}}^{3+}$ oxidation reaction, whereas at a low oxygen content it should approach the $\Delta H_{ox(2-3)}^{ο}$ = $\Delta H_{ox}^{ο}-2\Delta H_{d}^{ο}-\Delta H_{od}^{ο}$ value, which corresponds to the enthalpy of the $\frac{1}{2}O_{2}+2{\mathrm{Fe}}^{2+}+\left[V_{O2}^{}\right]⇄O_{O2}^{2-}+2{\mathrm{Fe}}^{3+}$ oxidation reaction. As for the partial molar entropy of oxygen, the behavior of this function, according to Equation (24), is conditioned mainly by the contribution of the configurational entropy. Since the partial molar enthalpy and entropy of oxygen are very sensitive to deviations in the oxygen content data, the general agreement of the experimental and calculated results indicates the correctness of the employed model that allows for considering the obtained defect concentrations reliable.

**Figure 10:**
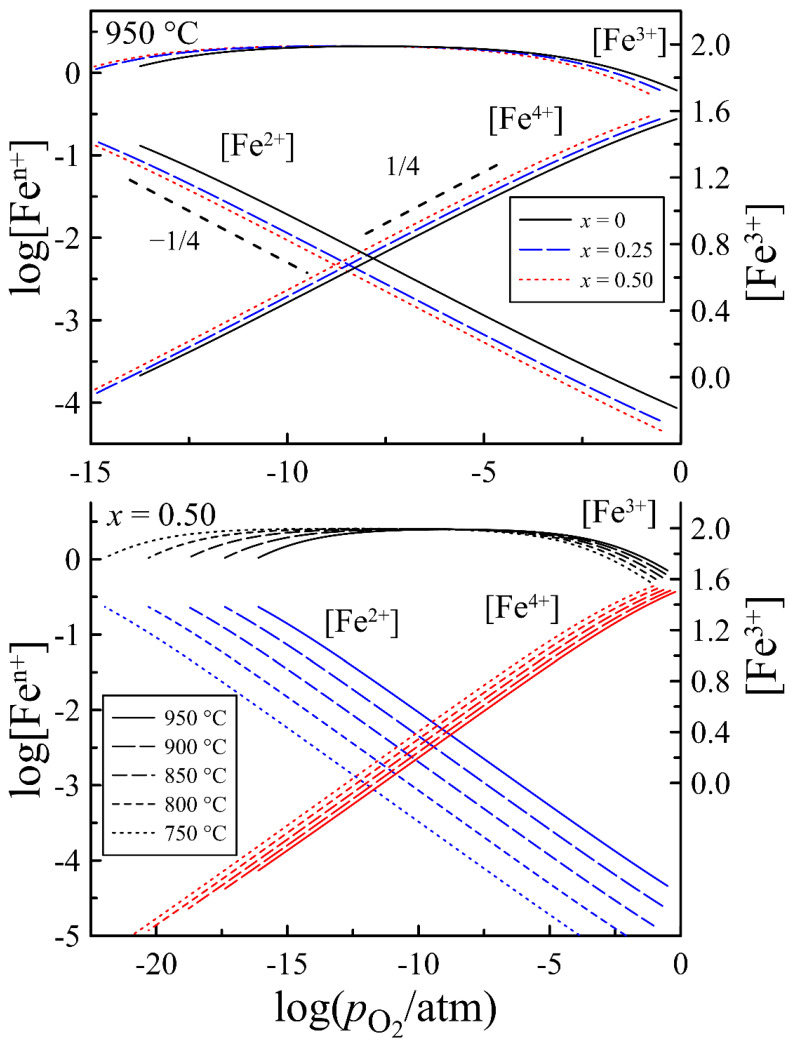
The concentration of iron ions in different oxidation states in PrBa_1−*x*_Sr*_x_*Fe_2_O_6−*δ*_ as a function of oxygen partial pressure at different temperatures.

The concentrations of iron ions calculated with the obtained thermodynamic parameters of defect formation reactions are shown in Figure 10. It can be observed that in wide ranges of $p_{O_{2}}$ the average oxidation state of iron ions is close to 3+, therefore the concentration of Fe^2+^ and Fe^4+^ is proportional to $p_{O_{2}}^{–1/4}$ and $p_{O_{2}}^{+1/4}$, respectively. The dependence of these concentrations on the ratio of barium and strontium is determined by the values of $\Delta H_{ox}^{ο}$ and $\Delta H_{ox(2-3)}^{ο}$. Comparison of the data in Table 1 and the curves in Figure 10 shows that the higher the values of $\Delta H_{ox}^{ο}$ and $\Delta H_{ox(2-3)}^{ο}$, the higher the concentration of Fe^4+^ and Fe^2+^ ions. In general, strontium substitution favors increasing the p-type carrier concentration.

**Figure 11:**
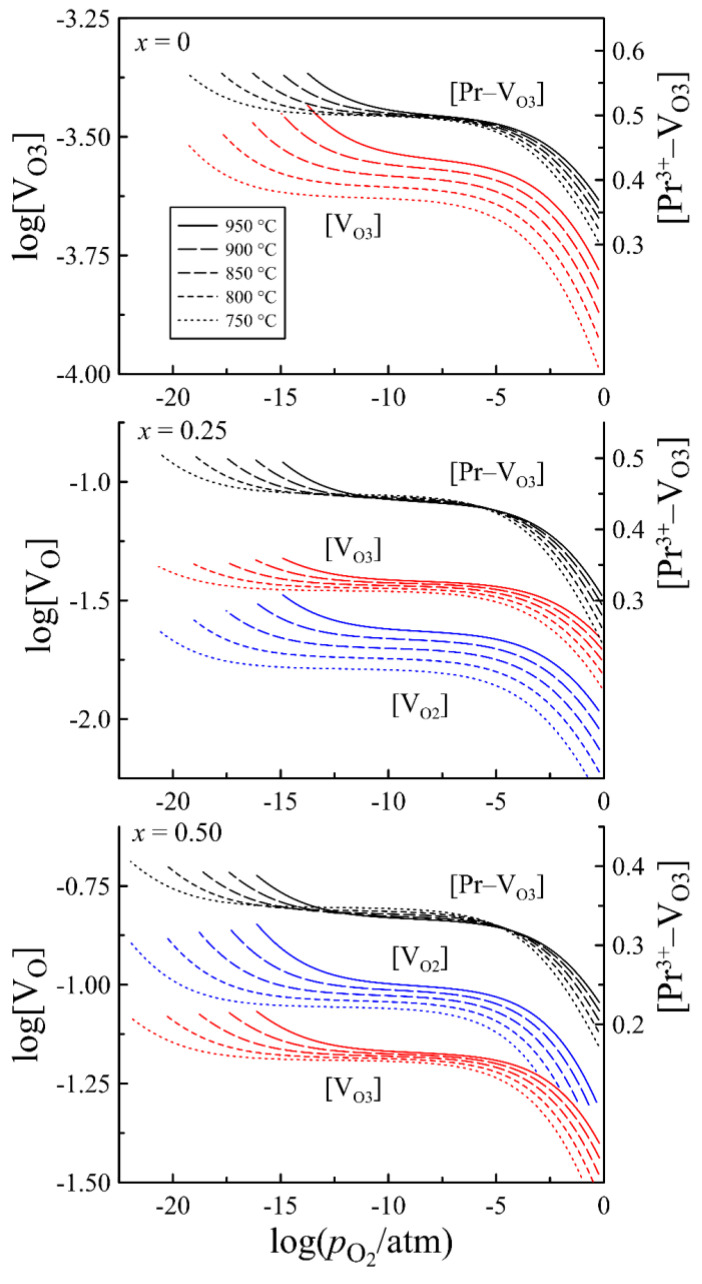
The concentration of free oxygen vacancies in O2 and O3 positions and clusters of [Pr^3+^–V_O3_] in PrBa_1−*x*_Sr*_x_*Fe_2_O_6−*δ*_ as a function of oxygen partial pressure at different temperatures.

A significant impact of strontium content on the defect formation in PrBa_1−*x*_Sr*_x_*Fe_2_O_6−*δ*_ is revealed in Figure 11, where oxygen vacancy distribution between available positions is shown. It can be observed that the main number of vacancies in all oxides are bound in clusters. A distinctive feature of the strontium-free composition is the absence of oxygen vacancies in the O2 positions and a very low concentration of free V_O3_ vacancies. Strontium substitution to the degree *x* = 0.25 results in two orders of magnitude increase in the concentration of free V_O3_ vacancies, and more importantly, in the appearance of oxygen vacancies in the O2 positions. At *x* = 0.5, the concentration of V_O2_ vacancies becomes higher than that of V_O3_. All of these indicate that the substitution of barium by strontium results in a progressive disordering of the anion sublattice in PrBa_1−*x*_Sr*_x_*Fe_2_O_6−*δ*_ that is in good agreement with the results of electron diffraction studies. The mechanism of strontium action can be assumed as follows. For small Pr^3+^ ions, the 12-fold oxygen coordination required for a complete anion sublattice is unfavorable, but a separate Pr^3+^ ion can acquire it under the force of the rest of the lattice. The ordering in A-sublattice caused by a big difference in size of Ba^2+^ (R_CN12_ = 1.610 Å) and Pr^3+^ (R_CN9_ = 1.179 Å) ions [29] collects Pr^3+^ ions into layers and enhances their ability to lower coordination. This promotes the ordering of the anion sublattice via the formation of steady clusters [Pr^3+^–V_O3_], and decreases the overall oxygen content in the oxide. Partial substitution of Ba^2+^ ions by smaller Sr^2+^ (R_CN12_ = 1.440 Å) [29], weakens the tendency to cation ordering, dissipates Pr^3+^ layers, destroys [Pr^3+^–V_O3_] clusters and results in the anion sublattice disordering (Figure 11) and in an increase in the oxygen content in the oxide (Figure 7). The higher oxygen content makes a higher concentration of p-type carriers according to reaction (1), as can be observed in Figure 10.

An increase in the concentration of free vacancies should favor oxygen transport, therefore partial substitution of barium by strontium can be considered as a promising approach to improve the operational characteristics of PrBaFe_2_O_6−*δ*_-based materials in electrochemical devices.

## 5. Conclusions

A series of PrBa_1−*x*_Sr*_x_*Fe_2_O_6−*δ*_ perovskite-type oxides were synthesized at 1350 °C. All oxides, both as-prepared and reduced at 950 °C and $p_{O_{2}}$ ~ 10^−15^ atm were characterized by XRD to have a cubic structure, whereas an electron diffraction study detected primary incommensurate modulations with wave vectors ***q*** = ±0.43$a^{*}$, which manifests a tendency to ordering in A—or/and anion sublattices.

The oxygen content in PrBa_1−*x*_Sr*_x_*Fe_2_O_6−*δ*_ was measured versus oxygen partial pressure in the interval between 10^−15^ and 0.5 atm at 750–950 °C by coulometric titration. The obtained data were used for the modeling of defect equilibrium in oxides. The point defect model assumed that the oxides consisted of chaotically oriented domains with an ordered double perovskite structure. Taking into account the reactions of oxygen vacancy distribution between non-equivalent positions, and their trapping in clusters with Pr^3+^ ions, in addition to the usual oxidation and charge disproportionation reactions, allowed for obtaining an appropriate agreement with the experimental data.

The modeling results have demonstrated that the partial substitution of strontium for barium in PrBa_1−*x*_Sr*_x_*Fe_2_O_6−*δ*_ leads to a progressive disordering of anion sublattice, an increase in the binding energy of oxygen in the oxides and an increase in the concentration of p-type carriers.

The correctness of the point defect model and the obtained thermodynamic parameters has been confirmed by a reasonably good agreement between the partial molar enthalpy and entropy of oxygen obtained from the experimental data and by statistical thermodynamic calculations.

## Figures and Tables

**Table 1:** Thermodynamic parameters of defect formation reactions in PrBa_1−*x*_Sr*_x_*Fe_2_O_6−*δ*_.

	*x* = 0	*x* = 0.25	*x* = 0.50
Δ*H*_ox_/kJ·mol^−1^	−80 ± 2	−82 ± 1	−85 ± 1
Δ*S*_ox_/J·mol^−1^K^−1^	−59 ± 2	−65 ± 1	−68 ± 1
Δ*H*_d_/kJ·mol^−1^	132 ± 4	133 ± 3	133 ± 3
Δ*S*_d_/J·mol^−1^K^−1^	10 ± 1	9 ± 1	8 ± 1
Δ*H*_od_/kJ·mol^−1^	–	15 ± 1	5.3 ± 0.6
Δ*S*_od_/J·mol^−1^K^−1^	–	−9.4 ± 0.9	−8.5 ± 0.7
Δ*H*_c_/kJ·mol^−1^	−10 ± 1	−7 ± 1	−5.7 ± 0.8
Δ*S*_c_/J·mol^−1^K^−1^	26 ± 2	19 ± 2	12 ± 2

## Data Availability

All data included in this study are available upon request by contact with the corresponding author.
